# Early Rehabilitation Reduces Time to Decannulation in Patients With Severe Acquired Brain Injury: A Retrospective Study

**DOI:** 10.3389/fneur.2018.00559

**Published:** 2018-07-10

**Authors:** Ilaria Zivi, Roberto Valsecchi, Roberto Maestri, Sara Maffia, Alessio Zarucchi, Katia Molatore, Elena Vellati, Leopold Saltuari, Giuseppe Frazzitta

**Affiliations:** ^1^Department of Brain Injury and Parkinson‘s Disease Rehabilitation, Ospedale Moriggia-Pelascini, Gravedona, Italy; ^2^Department of Intensive Care, Ospedale Moriggia-Pelascini, Gravedona, Italy; ^3^Department of Biomedical Engineering, Istituti Clinici Scientifici Maugeri Spa Società Benefit, IRCCS, Montescano, Italy; ^4^Research Unit for Neurorehabilitation South Tyrol, Landeskrankenhaus Hochzirl, Natters, Austria

**Keywords:** tracheostomy, decannulation, critical care, intensive care, neurorehabilitation, acquired brain injury, verticalization, early rehabilitation

## Abstract

**Purpose:** Early decannulation is considered a main rehabilitative goal in tracheostomized patients. Our aim is to evaluate whether a very early rehabilitation protocol helps to reduce the tracheostomy duration in patients affected by an Acquired Brain Injury (ABI).

**Methods:** Data about consecutive tracheostomized patients admitted in our Neuro-Rehabilitation Unit (NRU) were retrospectively collected. We defined two groups: Early Rehabilitation Group patients came from our ICU, where they started the rehabilitative treatment; Delayed Rehabilitation Group patients arrived from external ICUs and started rehabilitation in our NRU. Primary outcome was the time from tracheostomy to decannulation. Secondary outcomes were: ICU length of stay, time from NRU admission to decannulation, Glasgow Coma Scale, Coma Recovery Scale revised and Levels of Cognitive Functioning scores at NRU discharge and the re-cannulation rate.

**Results:** We enrolled 66 patients, 40 in the Early Rehabilitation Group and 26 in the Delayed Rehabilitation Group. 70% of patients for each group could be decannulated (*p* = 0.73) and were analyzed. Only one patient was re-cannulated. Early Rehabilitation Group showed a shorter tracheostomy duration (61.0 vs. 94.5 days, *p* = 0.013), a higher probability of occurrence of decannulation (*p* = 0.008) and a lower ICU length of stay (30.0 vs. 52.0 days, *p* = 0.001). The time to decannulation in NRU was similar between groups (30.0 vs. 45.50 days, *p* = 0.14). All the scale scores had a significant improvement in both groups (*p* < 0.0001 all).

**Conclusions:** The present study shows that an early neuro-rehabilitation protocol helps to reduce the time to decannulation in tracheostomized patients affected by ABI.

## Introduction

Tracheostomy is a commonly performed procedure in patients hospitalized in Intensive Care Unit (ICU) departments, mostly after the development of the safer percutaneous technique (1, 2). In patients with a low level of consciousness due to a severe Acquired Brain Injury (ABI) tracheostomy is usually placed when there is a need of more than 3 days of mechanical ventilation, in order to prevent laryngeal damages due to intubation, to prevent aspiration, to allow secretions suctioning and to lead to faster mechanical ventilation weaning and ICU discharge (1, 3–5).

Nevertheless, the presence of a tracheostomy tube seems to increase airflow resistance and work of breathing and to reduce air humidification and heating, thus facilitating mucosal alterations with consequent infections (6). Moreover, long-term tracheostomies were shown to produce obstructive airway complications (tracheal granuloma/stenosis, tracheomalacia, laryngeal lesions/disfunction) in up to 67% of the cases (7, 8) and to increase the rate of rehospitalization in respiratory patients (9). At last, the presence of a tracheal cannula impacts on patients' quality of life, affecting swallowing and phonation mechanisms, limiting their participation to the rehabilitation process and making the discharge home harder (10, 11). Therefore, an early but safe decannulation should be considered a main rehabilitative goal.

There is a broad variability in tracheostomy care and decannulation practices among the different centers: in absence of evidence-based guidelines, especially regarding ABI patients, they rely on the individual experience of the clinician and are affected by several environmental factors and chronic comorbidities (12, 13). In a recent review, Santus et al stated that the most frequent criteria used by clinicians to decide for decannulation are the ability to tolerate tube capping and the cough effectiveness. Less importance is given to oxygen saturation, secretions characteristics, level of consciousness, age, swallowing capability and comorbidities (6).

To date, little is known about the factors contributing to reduce the decannulation timing. According to several reports, a leading role is played by the presence of a multidisciplinary team involved in the tracheostomy care, that facilitates higher decannulation rates and shorter length of stay in the acute setting (14–16).

Aim of the present study was to evaluate whether a very early neuro-rehabilitative protocol started in ICU helps to reduce the decannulation time in patients affected by a severe ABI.

## Methods

We retrospectively evaluated consecutive patients affected by a severe ABI admitted in our Neuro-Rehabilitation Unit (NRU) over three years. Inclusion criteria: age>18 years, presence of a tracheal cannula at admission, tracheostomy performed for decreased mental status, arrival from an ICU. Exclusion criteria: tracheostomy performed before ICU admission and need for ventilation support.

Patients were divided into two groups, according to their arrival from our ICU or an external one. In both cases, patients are treated in ICU with the best medical and/or surgical treatment expected according to their particular etiology, comorbidities, secondary complications and clinical evolution.

All patients hospitalized in our ICU are evaluated by our Neurorehabilitation team and start a rehabilitative program in the first week. It lasts 60 min/day and is composed by in-bed mobilization, sensorial stimulations and sometimes stepping verticalization sessions with Erigo® (Hocoma, Switzerland), when meeting the inclusion/exclusion criteria previously reported (17, 18). Some of the patients enrolled in the present study had participated to our previous randomized controlled study about the effectiveness of an early stepping verticalization in ICU (18). The referring external ICUs do not provide rehabilitation.

As for our internal protocol, ABI patients are considered to be transferable in our NRU once they reach a clinical, neurological and radiological stability in ICU. At that time, they are enrolled in our unique waiting list for admission and are transferred in chronological order.

In NRU all patients receive an individualized multidisciplinary rehabilitative treatment. Consequently, rehabilitation is started since the acute phase of the ABI in patients arriving from our ICU (“Early Rehabilitation Group”); at admission in NRU (subacute phase) in patients arriving from external ICUs (“Delayed Rehabilitation Group”).

According to the rehabilitative treatment received in ICU, we differentiated a Verticalization Subgroup (conventional physiotherapy plus stepping verticalization sessions) and a Conventional Subgroup (only conventional physiotherapy).

As concerns our decannulation protocol in NRU, when the patient becomes alert, is able to tolerate tracheal tube capping for >24 h with the maintenance of a good oxygen saturation (SpO_2_ ≥ 95%) and has scarce quantity of secretions without need of suctioning (as for an effective cough mechanism), we perform a bronchoscopy. If it demonstrates the absence of an altered epiglottis and laryngeal motility, of tracheal anatomical anomalies and of secretions stasis in the lower airways, we proceed to decannulation.

We collected data about age, sex, etiology of the ABI, time from event to tracheostomy, Glasgow Coma Scale (GCS), Coma Recovery Scale revised (CRSr) and Levels of Cognitive Functioning (LCF) scores at NRU admission.

Primary outcome was tracheostomy duration (=days from tracheostomy to decannulation). Secondary outcomes were ICU length of stay, time to decannulation in NRU (= days from NRU admission to decannulation), the re-cannulation rate and the GCS, CRSr and LCF scores at NRU discharge.

For continuous variables, results were presented as mean ± SD or median (Q1-Q3) depending on the normality of the distributions, as assesses by the Shapiro–Wilk statistic. Number (frequency) were reported for categorical variables.

Between groups comparisons for continuous variables were performed by the Mann-Whitney *U*-test. Comparisons of categorical variables were carried out with the Chi-square test or Fisher exact test when appropriate.

The Kaplan-Meier method was used to estimate the probability of occurrence of decannulation at a certain point of time. Kaplan-Meier curves from the two groups were compared by the log-rank test.

Multivariable analysis of decannulation time was carried out using a Cox proportional hazards model, reporting hazard ratio with 95% confidence intervals.

The chosen level of statistical significance was 0.05. All analyses were performed using the SAS/STAT statistical package 9.2.

The study has been approved by the local ethics committee (“Comitato Etico Interaziendale di Como, Lecco e Sondrio”) and has therefore been performed in accordance with the ethical standards laid down in the Declaration of Helsinki, its later amendments and the national laws. The study was registered on ClinicalTrials.gov website (registration number NCT02990871). All patients or their legal surrogates had signed an informed consent for the use of their clinical data.

## Results

The study group consisted in 66 patients: 40 for the Early Rehabilitation Group and 26 for the Delayed Rehabilitation Group. No significant differences in age (*p* = 0.54), sex (*p* = 0.09) and etiology (*p* = 0.23) were observed in the two groups (Table 1). Table 2 reports the values of the scales considered at the admission in NRU, not different between groups. Eight patients (5 in Early Rehabilitation Group and 3 in the Delayed Rehabilitation Group, *p* = 0.91) deceased during hospitalization. Twenty-eight patients in the Early Rehabilitation Group (70%) and 18 in the Delayed Rehabilitation Group (70%) could be decannulated (*p* = 0.73) during their stay in the NRU and were analyzed. None of the 46 decannulated patients was intubated or re-cannulated during the entire NRU stay. One patient belonging to the Delayed Rehabilitation Group was transferred from NRU to ICU 9 days after the decannulation as result of the occurrence of a respiratory complication (pulmonary atelectasis) with the tracheal stoma still open. He was re-intubated and, after 33 days, re-tracheostomized (the previous tracheal stoma was stitched up because too close to the larynx, thus affecting the respiratory mechanics).

**Table 1:** Study population.

	**Early rehabilitation group (*N* = 40)**	**Delayed rehabilitation group (*N* = 26)**	***p*-value**
**Age (years)**	61.4 ± 15.7	58.2 ± 14.7	0.54
**Gender (M/F)**	25/15	22/4	0.09
**Etiology**			0.23
*Traumatic (N)*	10	3	
*Ischemic (N)*	4	2	
*Hemorrhagic (N)*	16	12	
*Anoxic (N)*	7	9	
*Other (N)*	3	0	

**Table 2:** Scale scores of the population at admission in NRU.

**Variable**	**Early rehabilitation group (*N* = 40)**	**Delayed rehabilitation group (*N* = 26)**	***p*-value**
GCS	10.2 ± 3.5	9.1 ± 2.8	0.17
CRSr	11.2 ± 7.6	8.5 ± 5.4	0.19
LCF	3.1 ± 1.6	2.6 ± 1.3	0.33

*GCS, Glasgow Coma Scale; CRSr, Coma Recovery Scale revised; LCF, Levels of Cognitive Functioning*.

The tracheostomy duration was shorter in the Early Rehabilitation Group [61.0 (31.0, 91.0) vs. 94.5 (57.0, 155.0) days, *p* = 0.013] while the time from event to tracheostomy was comparable in the two Groups [7.5 (5.0, 13.0) vs. 6.5 (5.0, 10.0) days, *p* = 0.47].

**Figure 1:**
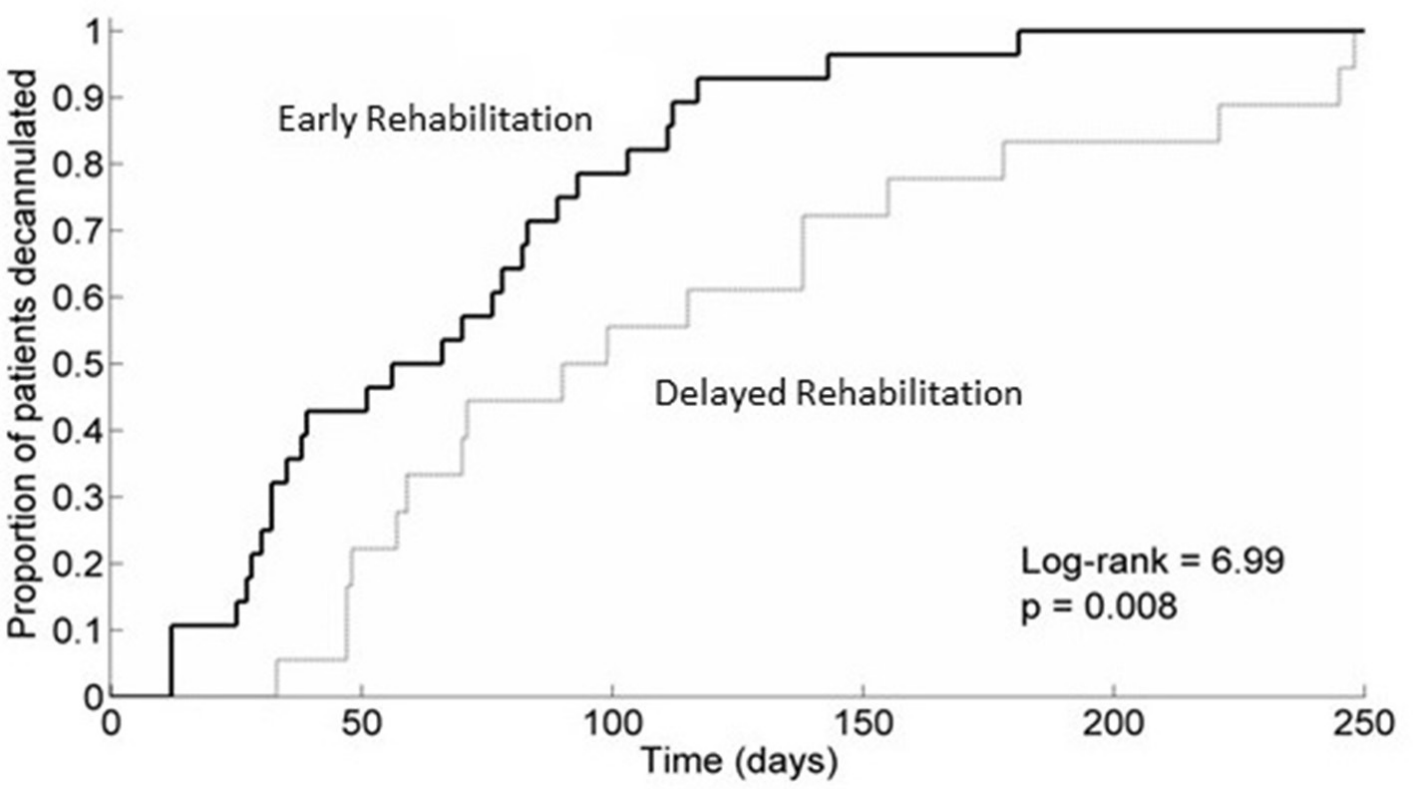
Kaplan-Meier analysis of the probability of decannulation in the two groups. The figure shows a higher probability of occurrence of decannulation at a certain point of time in the Early Rehabilitation Group. Group 1, Early Rehabilitation Group; Group 2, Delayed Rehabilitation Group.

Kaplan-Meier analysis (Figure 1) showed that the probability of occurrence of decannulation at a certain point of time was higher in the Early Rehabilitation Group than in the Delayed Rehabilitation Group (*p* = 0.008). Multivariable Cox analysis revealed that the chance of decannulation increased by more than 300% for patients in the Early Rehabilitation Group, (hazard ratio = 4.095, 95% confidence intervals = 1.427, 11.752), after correction by age, etiology and clinical characteristics at admission in NRU.

The time to decannulation in NRU was similar between groups [30.0 days (10.5, 62.0) vs. 45.50 days (21.0, 113.0), *p* = 0.14]. The ICU length of stay was significantly lower in the Early Rehabilitation Group [30.0 days (23.0, 39.5) vs. 52.0 days (32.0, 68.0), *p* = 0.001].

Table 3 reports the values of considered scales at NRU discharge. All variables improved in both groups respect to NRU admission (*p* < 0.0001 all). Finally, considering patients in the Early Rehabilitation Group subdivided according to having or not received stepping verticalization sessions in ICU (Verticalization Subgroup, *n* = 13 and Conventional Subgroup, *n* = 15, respectively), a clear trend toward a shorter decannulation time in the Verticalization Subgroup was observed [39.0 (24.0, 77.5) vs. 78.0 (33.5, 100.5) days] but statistical significance was not reached (*p* = 0.22). In Table 4 the values of considered scales at NRU discharge in the two subgroups are reported: no significant differences were observed in the considered variables, but a trend toward a better status in the Verticalization Subgroup was noted. Indeed, comparing the two subgroups individually with the Delayed Rehabilitation Group at NRU discharge, no differences were observed between the Conventional Subgroup and the Delayed Rehabilitation Group, while decannulation time was better in the Verticalization Subgroup than in the Delayed Rehabilitation Group (*p* = 0.012).

**Table 3:** Scale scores of the population at NRU discharge.

**Variable**	**Early rehabilitation group (*N* = 28)**	**Delayed rehabilitation group (*N* = 18)**	***p*-value**
GCS	15.0 (12.0,15.0)	15.0 (13.0,15.0)	0.76
CRS	23.0 (9.5,23.0)	20.5 (16.0,23.0)	0.71
LCF	6.00 (3.00,7.00)	5.50 (5.00,7.00)	0.68

*GCS, Glasgow Coma Scale; CRSr, Coma Recovery Scale revised; LCF, Levels of Cognitive Functioning*.

**Table 4:** Scale scores of Early Rehabilitation subgroups at NRU discharge.

**Variable**	**Verticalization Subgroup (*N* = 13)**	**Conventional Subgroup (*N* = 15)**	***p*-value**
GCS	15.0 (12.8,15.0)	14.5 (12.0,15.0)	0.46
CRS	23.0 (17.0,23.0)	18.0 (9.0,23.0)	0.32
LCF	7.00 (5.00,7.00)	5.00 (3.00,6.00)	0.29

*GCS, Glasgow Coma Scale; CRSr, Coma Recovery Scale revised; LCF, Levels of Cognitive Functioning*.

## Discussion

The present study shows that an early neuro-rehabilitation protocol helps to reduce the time to decannulation in tracheostomized patients affected by ABI. This result emphasizes the known importance of the integration of a rehabilitative treatment in the ICU care of this group of patients, in terms of better functional outcome (19–21).

To our knowledge, this is the first study that assesses the effects of a neuro-rehabilitation program started in ICU on the decannulation timing of patients affected by severe ABI.

Our two groups were similar according to age, sex, main scale scores at NRU admission (GCS, CRSr and LCF), mortality rate and number of decannulated patients during NRU stay. This homogeneity emphasizes the role that the rehabilitation timing (acute vs. subacute phase), main discriminating factor between the two groups, plays in determining possible outcomes differences.

As regards the primary outcome, we found that patients who received a neuro-rehabilitative treatment in ICU, compared to the ones who started rehabilitation once admitted in NRU, showed a significant shorter tracheostomy duration, being decannulated faster.

We considered early decannulation as a main issue because it may help to avoid secondary complications such as respiratory infections and airway obstructions (6–8) and to improve patients' quality of life and rehabilitation participation (10, 11). Patients coming from our ICU do not receive respiratory rehabilitation before admission in NRU, but only sensorial stimulation and mobilization exercises with or without verticalization sessions. Their better outcome in terms of tracheostomy duration may be due to the shorter bed rest, which is known to affect inflammation processes, neuromuscular activity and pulmonary function (19).

The shorter ICU length of stay of the Early Rehabilitation Group underlines how decisive the administration of an early rehabilitation is even on the intensive care weaning of ABI patients. This result confirms previous reports about the importance of an early multidisciplinary intervention on patients with ABI, in order to shorten the ICU stay and avoid the physical deconditioning, thus optimizing patients' functional outcomes and recovery potential (19–23).

The similar time to decannulation in NRU and the significant improvement of the scale scores (GCS, CRSr, LCF) between NRU admission and discharge in both groups prove the absence of a difference in the rehabilitative approach that the patients received during the NRU phase, isolating the importance of the early rehabilitation and emphasizing the groups homogeneity.

When considering different early rehabilitative approaches, we observed that patients who received stepping verticalization sessions in ICU had a trend toward a more favorable outcome in terms of decannulation time and neurological status (scale scores at discharge) than patients who received only conventional physiotherapy. The absence of a statistical significance is likely due to the small sample size, but the result is in line with our previous finding about the efficacy of a very early stepping verticalization treatment on the functional and neurological outcome of ABI patients (18). This better improvement may be due to the sensorial stimulation in orthostatic position (24) and to the shortness of the head-down bed rest, not enough to alter the autonomic and endocrine functions (25).

Nevertheless, as retrospective study with a relative small sample size, our findings have not the power to give definite conclusions about the topic, but they can represent a first description encouraging the planning of larger multicenter clinical trials. According to the literature indications, we consider the rehabilitation treatment a keystone in the care of brain injured patients since their acute phase, able to take advantage of the positive neuroplasticity mechanisms when they are at their maximum and to avoid physical deconditioning with secondary complications. For this reason, in our center early rehabilitation is routinely part of the patients' care in NeuroICU and is performed in all admitted patients since the first week from the event.

Main limit of the study is the heterogeneity of origin of the population, that could have influenced different patient managements in the acute phase. However, each sending ICU has operated following the “best medical treatment” criteria and the outcome scales at admission in NRU (corresponding to the ICU discharge) showed no substantial difference in patients' neurological state between the two groups. The two populations, even if coming from different ICUs, are indeed homogeneous at NRU admission: the heterogeneity of the clinical evolution in the acute phase can indeed affect all patients, irrespective of the hosting acute ward. Despite that, both groups of patients arrived in NRU in the same clinical and neurological conditions and carrying a tracheal cannula. Given that our NRU manages a unique waiting list, which considers as inclusion criterion for admission only the patients' stability, patients' transfer from any ICU to our NRU does not follow different pathways according to the origin, but only a chronological order, with a mean waiting time of 10–15 days according with the bed availability.

In conclusion, despite the cited limits and bearing in mind the literature findings, we argue that the found differences between the two groups in terms of decannulation timing and length of stay in ICU should be considered as subject to the different rehabilitation timings.

## Data availability

The raw data supporting the conclusions of this manuscript will be made available by the authors, without undue reservation, to any qualified researcher.

## Author contributions

IZ and GF: conception of the study, interpretation, writing and revision guarantors; RV and LS: conception of the study and revision; SM, AZ, KM, and EV: acquisition and collection of data; RM: analysis of data, writing, and revision.

### Conflict of interest statement

The authors declare that the research was conducted in the absence of any commercial or financial relationships that could be construed as a potential conflict of interest.
